# Comparative transcriptomics identifies patterns of selection in roses

**DOI:** 10.1186/s12870-018-1585-x

**Published:** 2018-12-22

**Authors:** Shubin Li, Micai Zhong, Xue Dong, Xiaodong Jiang, Yuxing Xu, Yibo Sun, Fang Cheng, De-zhu Li, Kaixue Tang, Siqing Wang, Silan Dai, Jin-Yong Hu

**Affiliations:** 1 Beijing Key Laboratory of Ornamental Plants Germplasm Innovation & Molecular Breeding, National Engineering Research Center for Floriculture, Beijing Laboratory of Urban and Rural Ecological Environment, Key Laboratory of Genetics and Breeding in Forest Trees and Ornamental Plants of Ministry of Education, School of Landscape Architecture, Beijing Forestry University, 35 East Qinghua Road, Beijing, 100083 China; 2 Group of Plant Molecular Genetics and Adaptation, CAS Key Laboratory for Plant Diversity and Biogeography of East Asia, Kunming Institute of Botany, Chinese Academy of Sciences, Kunming, 650201 China; 3 University of Chinese Academy of Sciences, Beijing, 100049 China; 4 Germplasm Bank of Wild Species, Kunming Institute of Botany, Chinese Academy of Sciences, Kunming, 650201 China; 5 Flower Research Institute, Yunnan Agricultural Academy of Sciences, Kunming, 650231 China

**Keywords:** *Rosa sp*., Selection pattern, Comparative transcriptomics, Rosaceae-common, *Rosa*-specific

## Abstract

**Background:**

Roses are important plants for human beings with pivotal economical and biological traits like continuous flowering, flower architecture, color and scent. Due to frequent hybridization and high genome heterozygosity, classification of roses and their relatives remains a big challenge.

**Results:**

Here, to identify potential markers for phylogenetic reconstruction and to reveal the patterns of natural selection in roses, we generated sets of high quality and comprehensive reference transcriptomes for *Rosa chinensis* ‘Old Blush’ (OB) and *R. wichuriana* ‘Basye’s Thornless’ (BT), two species exhibiting contrasted traits of high economical importance. The assembled reference transcriptomes showed transcripts N50 above 2000 bp. Two roses shared about 10,073 transcripts (N50 = 2282 bp), in which a set of 5959 transcripts was conserved within genera of *Rosa*. Further comparison with species in Rosaceae identified 4447 transcripts being common (Rosaceae-common) in *Rosa, Malus*, *Prunus*, *Rubus*, and *Fragaria*, while a pool of 164 transcripts being specific for roses (*Rosa*-specific). Among the Rosaceae-common transcripts, 409 transcripts showed a signature of positive selection and a clustered expression in different tissues. Interestingly, nine of these rapidly evolving genes were related to DNA damage repair and responses to environmental stimulus, a potential associated with genome confliction post hybridization. Coincident with this fast evolution pattern in rose genes, 24 F-box and four TMV resistant proteins were significantly enriched in the *Rosa*-specific genes.

**Conclusions:**

We expect that these Rosaceae-common and *Rosa*-specific transcripts should facilitate the phylogenetic analysis of Rosaceae plants as well as investigations of *Rosa*-specific biology. The data reported here could provide fundamental genomic tools and knowledge critical for understanding the biology and domestication of roses and for roses breeding.

**Electronic supplementary material:**

The online version of this article (10.1186/s12870-018-1585-x) contains supplementary material, which is available to authorized users.

## Background

Understanding the molecular mechanisms underlying the adaptation of woody plants to local environmental conditions remains a big challenge in biology due to their long and perennial life history. However, woody plants represent a large proportion of biodiversity on the earth and harbor many different phenological traits that herbaceous plants do not feature (https://www.worldwildlife.org). One such trait is the continuous flowering behavior of rose, an important crop of high importance in human society. Guaranteeing a constant supply of raw materials for cut flowers and related products, continuous flowering becomes one of the most important biological and economical traits for roses [1]. Therefore, the genetic control and related gene-regulatory-network for continuous flowering regulation attracts efforts for many years not only from scientists but also from breeders [1, 2]. The number of QTL regulating continuous flowering remains disputable [1–3]. *RoKSN,* a homolog of Arabidopsis *TFL1*-like gene in roses, is the only known gene responsible for continuous flowering [4, 5].

Domestication of cultivated roses mainly involves hybridization among more than a dozen species [2, 6–8]. Frequent inter-species crossing/backcrossing and polyploidization of roses has made the classification of roses very difficult [9–12]. A set of high-quality and well-characterized genomic tools/resources are necessary for understanding the biology and domestication of modern roses that encompass more than 30,000 cultivars [13]. Recently, several genetic mapping populations have been developed (see reviews [1, 2]) and determination of rose genomes is carried out [14] with the sequence released very recently for a doubled-haploid of *Rosa chinensis* ‘Old Blush’ [15]. However, due to high-level heterozygosity caused very likely by inter-species crossing and polyploidization, achieving an accurate and complete rose genome seems not so easy. Alternatively, a comprehensive gene expression atlas can be constructed with multiple tissues in different species.

The first sets of gene expression atlas were constructed using microarrays containing about 350 (tetraploid *R. hybrida*) [16] and later with about 4800 selected ESTs (*R. chinensis*, *R. wichuriana*, and *R. hybrida*) [17]. A more comprehensive database containing about 80,714 transcript clusters for *R. chinensis* ‘Old Blush’ was constructed from 13 tissues/organs at different developmental stages or under different abiotic and biotic stresses with Illumina and 454 sequencing platforms [18]. Several recent studies have also been done with various *Rosa* species for different purposes [19–22] even for a single-nucleotide-polymorphism (SNP) array [23, 24]. Though all these studies have promoted significantly the understanding of the rose biology, the quality of these transcriptomes is normally relatively poor with low N50 value, poor completeness, and short average length. Very recently the publishing of genome sequences of a doubled-haploid for *R. chinensis* ‘Old Blush’ set a milestone for rose research [15, 25]. However, missing half genome information of roses, most of which often feature high heterozygosity due to frequent intra−/inter-species hybridization and polyploidization, might cause lacking of power in understanding the genetic bases of roses traits associated with recessive markers [3].

To identify the molecular components underpinning rose specialty and to find molecular markers for clarifying phylogenetic relationship of Rosaceae, we generated a set of high quality reference transcriptomes for two rose species, which featured at least six pair of contrast traits [3], by sequencing three tissues at different developmental stages and by integrating published datasets. We identified about 4447 transcripts conserved in Rosaceae plants, among which 405 were under significant selection pressure, and 164 transcripts present only in roses.

## Results

### Transcriptome sequencing and assembling

**Fig. 1:**
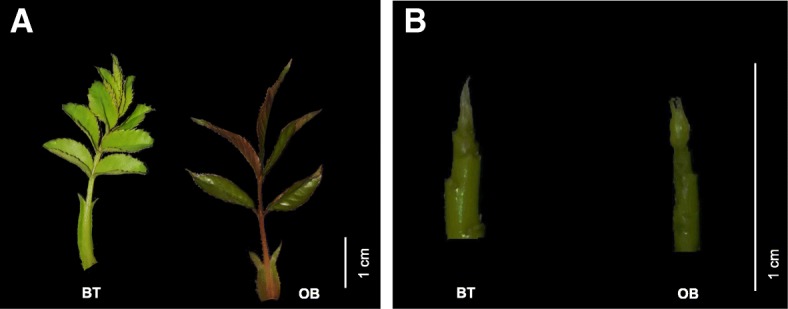
Leaf (**a**) and shoot (**b**) materials used for RNA-seq in this study. For each panel, left for *Rosa wichuriana* ‘Basyes’ Thornless’ (BT), and right for *R. chinensis* ‘Old Blush’ (OB). Bars = 1 cm

**Fig. 2:**
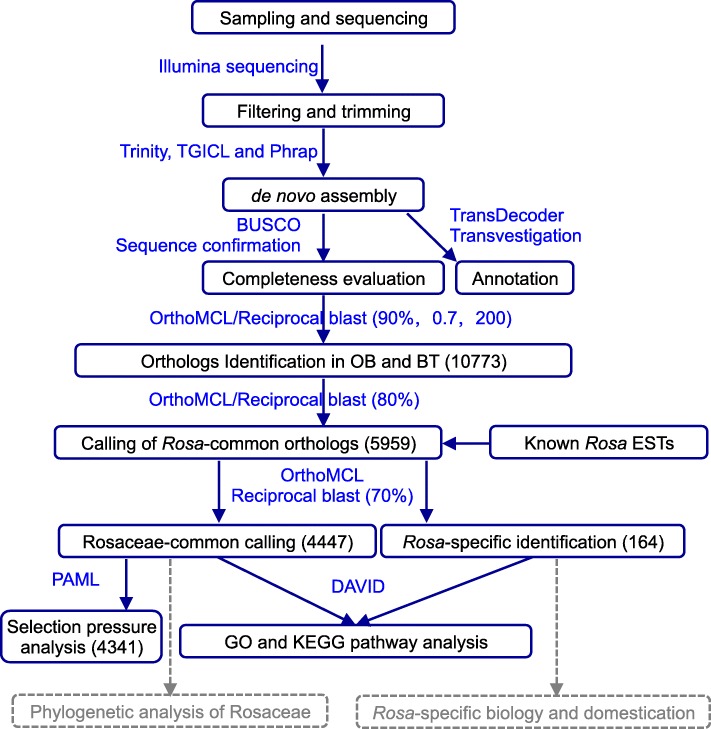
Working flow for assembling of reference trancriptomes and identification of Rosacaeae-common and *Rosa*-specific transcripts. Main steps were shown in boxes with key transcripts numbers given. Major tools used in these analysis were marked in blue. Dashed arrows and boxes indicated the data generated from this study could be explored in these applications

RNA samples from shoot materials and young leaves were profiled with high-throughput sequencing (Figs. 1 and 2). This resulted in 421.8 million and 427.5 million clean reads for *Rosa chinensis* ‘Old Blush’ (OB) and *R. wichuriana* ‘Basye’s Thornless’ (BT), respectively (Table 1). The raw sequence files have been uploaded to the National Center for Biotechnology Information Sequence Read Archive (http://www.ncbi.nlm.nih.gov/sra/) under the accession numbers SAMN07808857–07808870.

**Table 1:** Summary of sequencing strategies and sequences obtained

Species	Sample	Repetition	Reads number	Reads bases (nt)	Q20 (%)	Accession code
*Rosa wichuriana* ‘Basye’s Thornless’ (BT)	SAM^a^	Total	156,722,692	14,104,983,240	95.8	_
		Rep1	51,669,692	4,650,272,280		SAMN07808870
		Rep2	53,341,618	4,800,745,620		SAMN07808871
		Rep3	51,710,726	4,653,965,340		SAMN07808872
	leaf_nov^b^	Total	131,025,452	18,923,248,232	95.8	_
		Rep1	42,294,066	6,100,424,928		SAMN07808867
		Rep2	42,725,012	6,170,189,794		SAMN07808868
		Rep3	46,006,374	6,652,633,510		SAMN07808869
	leaf_mar^b^	Total	134,110,978	19,389,814,312	96.1	_
		Rep1	44,236,512	6,418,203,960		SAMN07808864
		Rep2	46,651,880	6,762,697,476		SAMN07808865
		Rep3	43,222,586	6,208,912,876		SAMN07808866
*Rosa chinensis* ‘Old Blush’ (OB)	SAM^a^	Total	159,834,774	14,385,129,660	95.3	_
		Rep1	52,443,532	4,643,438,236		SAMN07808861
		Rep2	53,284,740	4,800,745,624		SAMN07808862
		Rep3	54,106,502	4,940,945,800		SAMN07808863
	leaf_nov^b^	Total	137,399,212	19,719,505,590	95.8	_
		Rep1	44,950,340	6,501,086,190		SAMN07808858
		Rep2	45,260,556	6,426,857,278		SAMN07808859
		Rep3	47,188,316	6,791,562,122		SAMN07808860
	leaf_mar^b^	Total	130,341,518	18,669,073,728	96.7	_
		Rep1	42,299,982	6,021,996,256		SAMN07808855
		Rep2	43,001,278	6,170,274,204		SAMN07808856
		Rep3	45,040,258	6,476,803,268		SAMN07808857
	all other data^c^		550,108,308	63,356,156,640	95.6	_

Data are sum of three biological replications. ^a^and ^b^, samples sequenced via Illumina pair-end methods (PE100bp for ^a^ and PE150bp for ^b^);^c^, data from references (see Table 2)

**Fig. 3:**
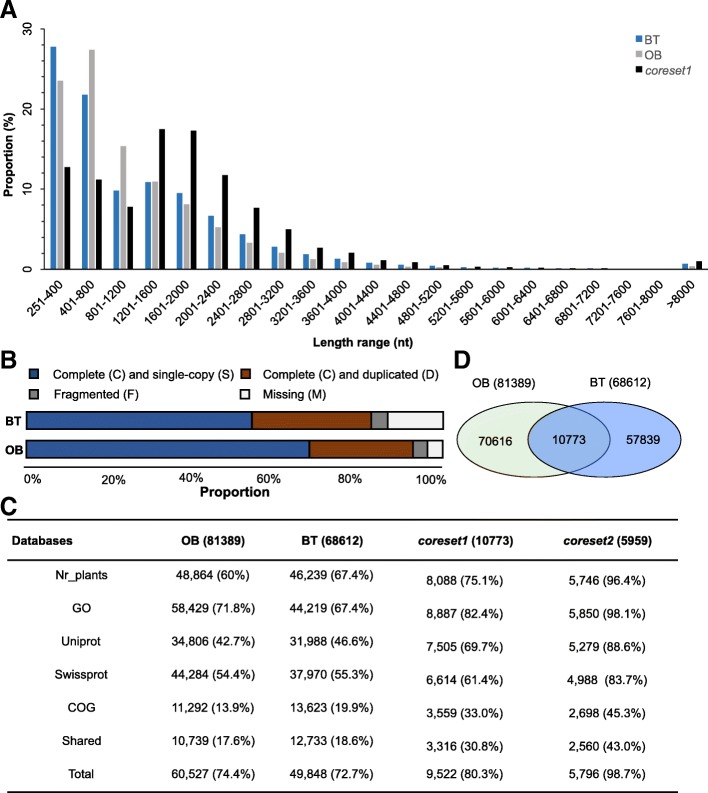
The assembly of high quality transcriptomes for roses. **a** Length distribution in proportion of assembled unigenes for the two species, *Rosa chinensis* ‘Old Blush’ (OB, bars filled in grey color), and *R. wichuriana* ‘Basyes’ Thornless’ (BT, open bars). Bars filled in black color mark the length distribution of shared transcripts between the two species (*coreset1*; see below and main text). **b** BUSCO analysis shows the completeness of assemblies and *coreset1*. **c** Annotation results of the assembled unigenes and *core-set*s for *Rosa*. The *coreset1* is between the two species, while *coreset2* is for the unigenes shared among *Rosa* (see Fig. 2) based on published and newly collected data from this study. For each category (Nr_plants, GO, Uniprot, Swissprot and COG databases), total unigene counts annotated in different databases besides the proportion (in brackets) are given. Shared and total unigenes annotated by all databases are also given. **d** Venn diagram shows the results of *coreset1* identification. About 10,773 transcripts were identified at the 95% sequence identity level between the two species

Next we generated the assemblies for BT (68,612 transcripts) and OB (74,975 transcripts) with transcripts N50 about 2099/1732 bp (BT/OB) and average length about 1338/1170 bp (BT/OB). This was much better than previously published data for these two species (Table 2, Additional file 1: Table S1, Additional file 2: Table S2; Fig. 3a) [18, 20, 26, 27] and other species/materials in *Rosa* [19, 21, 22, 28]. A further assembly with all available data for OB produced even better transcript N50 (2092 bp) and average length (1359 bp). Comparing the assembled OB transcripts with the genome assembly (v2.0_a1) that containing 45,469 coding and 4918 non-coding genes [15] revealed that almost 100% of these transcripts could be mapped. On the other hand, by looking for the genes included in the genome assembly in the assembled transcripts we identified about 97% of these genes in our OB assemblies. Mean GC content of all assemblies (44.2–46.4%) was comparable to that of published (45.8–46.5%) roses (Table 2). A BUSCO analysis revealed a high proportion of complete (C) and single copy (S) from 54.4 to 68.8%, and complete (C) and duplicated (D) from 24.5 to 28.2%. Fragmented (F) and missing (M) BUSCO items occupied about 5.6–17.4% (Fig. 3b; Additional file 1: Table S1). The prediction of high number of transcripts could be correlated with high frequency of intra−/inter-species hybridization and polyploidization in roses. Taken together, these results suggest the high quality, completeness, and coverage of the assembled BT and OB transcriptomes.

**Table 2:** Statistics of final assemblies for this study and published data

Assembly components	Contig number	Transcript number	Transcript N50	GC content (%)	Total assembled bases	Average length (bp)	Data sources
BT^a^	86,642	68,612	2099	46.4	92 M	1338	This study
OB^a^	99,456	81,389	2092	44.2	111 M	1359	This study
OB	na.	80,714	na	na.	36 M	444	Dubois et al. 2012 [18]
OB^b^	na.	68,565	na.	46.46	61 M	887	Yan et al. 2016 [20]
OB	na	85,663	na	na.	70 M	814	Guo et al. 2017 [26]
OB^c^	208,039	111,954	1997	45.8	231M^c^	1111	Han et al. 2017 [27]
Core set 1 BT vs. OB; 90% identity	na.	10,773	2282	na.	20 M	1863	This study
*R. multiflora*	78,676	61,864	1907	46.03	75 M	1216	Zhang et al. 2016 [21]
*R. jacq* cv. gold medal	na.	80,226	na	na.	60 M	743	Gao et al. 2016 [22]
*R. roxburghii*	na.	106,590	na	na.	37 M	343	Yan et al. 2015 [28]
*R. hybrida*	93,947	na.	1589	na.	na.	na.	[22]
*R. chinensis ‘*pallida’	na.	89,614	Na.	na.	38 M	428	Yan et al. 2014 [19]
*Rosa* transcriptome^d^	60,944	na.	314	na.	18 M	302	Fei lab
Core set 2 All samples; 80% identity	na.	5959	2326	na.	13 M	2161	This study

^a^assembly based on data produced from this study; ^b^, assembly based on data from this study and references Yan et al. [20] and Han et al. [27]; ^c^, conceptual confusion in original text; ^d^, data from Fei lab (http://bioinfo.bti.cornell.edu/cgi-bin/rose_454/index.cgi) with transcript N50 and average length recalculated

### Functional annotation

Five databases, including NR, GO, Uniport, Swissprot and COG databases, were explored to annotate the function of these assemblies. About 72.7% (BT) to 74.4% (OB) transcripts could be annotated with either database, while only about 17.6% (OB) to 18.6% (BT) transcripts with annotation shared for all databases (Table 2). Interestingly, about 67.4% (BT) to 71.8% (OB) transcripts features GO annotation, making the GO enrichment analysis feasible in following steps. Detailed annotation information was included in Fig. 3c as well as Additional file 2: Table S2 and Additional file 3: Figure S1. For both species, categories related to *Fragaria* presented the biggest proportion of transcripts (Additional file 2: Table S2).

### Identification of the conserved orthologous transcript elements sets between OB and BT (*coreset1*), and for *Rosa* (*coreset2*)

With an aim at identifying the transcripts shared for all Rosaceae plants, we first identified the transcripts shared between the two species. With a relatively stringent threshold (identity = 90%, minimum length coverage =0.7, minimum alignment length = 200 bp) we identified 10,073 unique transcripts shared by the two species (*coreset1*; Fig. 3d and Table 2). Interestingly, *coreset1* showed more than 80% of the transcripts with annotation (Fig. 3c and Additional file 4: Table S3).

We next screened for the transcripts common for all the *Rosa* plants with sequence identity set at 80%. With this threshold, about 5959 transcripts (N50 at 2326 bp and mean length at 2161 bp) were shared in all *Rosa* species (*coreset2*) with most of them well annotated (98.7%; Table 2; Fig. 3c).

### Identification of the Rosaceae-common and *Rosa*-specific transcripts

**Fig. 4:**
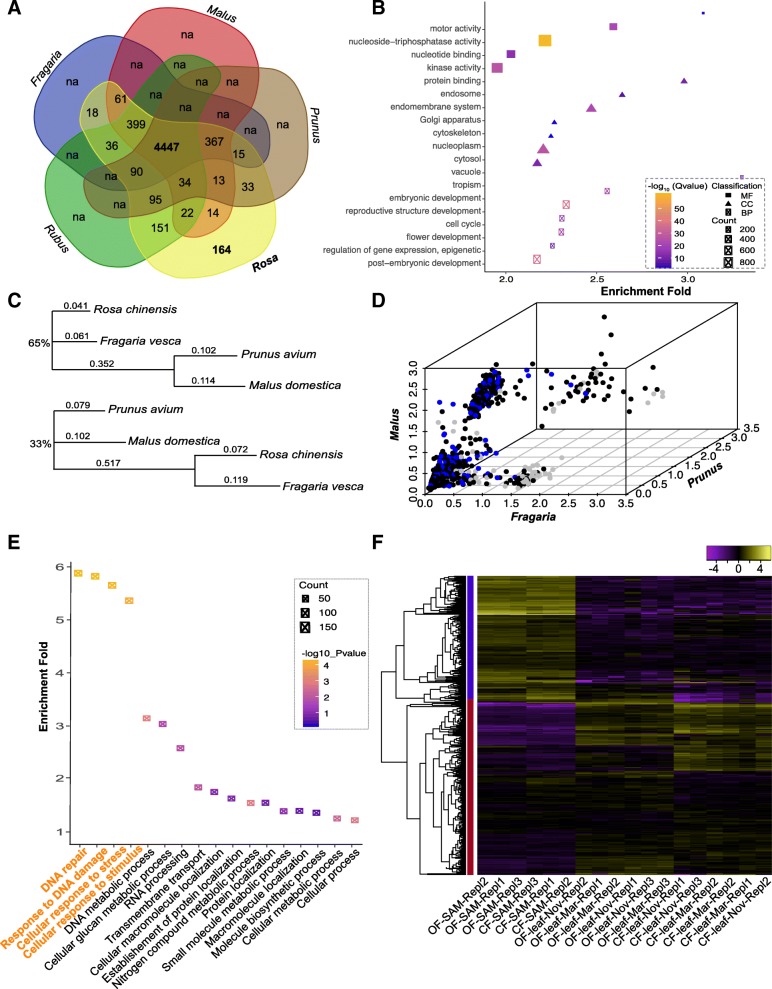
Identification and characterization of Rosaceae-common potential coding gene. **a** Venn diagram shows the Rosaceae-common and *Rosa*-specific transcripts. Note that, except *Rosa*, transcripts specific for other genera were not identified (marked with na). For that we are not interested in other share sets. **b** GO enrichment analysis of the 4447 Rosaceae-common transcripts (http://bioinfo.cau.edu.cn/agriGO). X-axis shows the enrichment fold of specific GO terms in comparison with the background. BP, CC and MF mean biological process, cellular component and molecular function separately. The area indicates gene counts. **c** Representive phylogentic topologies based on 4447 Rosaceae-common genes. Upper panel indicates about 65% topologies (2812) supporting the clustering of *Prunus* with *Malus*, while the topology in lower panel is supported by 33% genes (1436). Numbers on branches indicate distance. **d** Three-dimensional plots for the genetic distances of the 4447 transcripts between *Rosa* and *Fragaria* (X-axis) /*Malus* (Y-axis) /*Prunus* (Z-axis). Black and blue dots mark the genes supporting the topologies in C (Black for upper panel and blue for lower panel), while gray dots show genes supporting other topologies. **e** Distribution and GO enrichment analysis of the 409 selected Rosaceae-common transcripts. Y-axis shows the enrichment fold of specific GO terms in comparison with the background. Only four GO items are significantly enriched (marked in orange color). **f** Clustered heat map comparing scaled expression values for the 409 selected Rosaceae-common transcripts. Yellow indicates higher while purple marks lower expression. Blue and red bars indicate membership in the identified transcription clusters

One of the purposes of this research was to find transcripts/markers that could be potentially used for phylogenetic reconstruction of Rosaceae plants. We then compared the *coreset2* transcripts with genes from *M. domestica, R. occidentalis, P. avium,* and *Fragaria veseca*, the closest relatives of *Rosa* (Fig. 4a). About 4447 transcripts were found to present in all five genus (Rosaceae-common; 74.6% of *coreset2* transcripts), while only 164 transcripts were specific for *Rosa* plants (*Rosa*-specific; 2.75% of *coreset2*). Most of the Rosaceae-common transcripts were annotated (4228 or about 96%; Table 2 and Additional file 5: Table S4) and could encode for proteins (4341; 97.6%). About 504 Rosaceae-common transcripts belong to the 1440 BUSCO single-copy genes (35%; Additional file 5: Table S4, Additional file 6: Table S5). GO enrichment assay revealed that most of these transcripts were involved in very important and basic function (Fig. 4b, Additional file 5: Table S4). Phylogenetic analysis showed that most of Rosaceae-common transcripts supported the clustering of *P. avium* with *M. domestica* (2812 or 65%; Fig. 4c upper panel and Fig. 4d black dots) or *R. chinensis* with *F. veseca* (1436 or 33%; Fig. 4c lower panel and Fig. 4d blue dots) (Additional file 7: Figure S2).

### Features of the Rosaceae-common transcripts

Among the 4447 Rosaceae-common transcripts, 4341 (97.6%) had the coding potential and were used for selection pressure analysis with the ratio of *dN* (non-synonymous) vs. *dS* (synonymous) changes. *Malus/Prunus/Fragaria* orthologous sequences were taken as background (Additional file 8: Table S6). This analysis identified 409 transcripts significantly selected with *P* < 0.05 (after *Bonferroni correction*). Surprisingly, about 42% these genes (173) displayed a higher expression in shoot apical meristems (SAM; Fig. 4f, blue bar), while the expression of the others being high in leaf of both species or lower expressed in both SAM and leaf materials (Fig. 4f, red bar). These significantly selected genes showed an enrichment GO items for DNA repair (GO:0006281), responses to DNA damages stimulation (GO:0006974), cellular response to stress (GO:0033554) and cellular response to stimulus (GO:0051716) (Fig. 4e, Additional file 9: Table S7). Interestingly, all four GO items featured the same set of nine genes (Additional file 10: Table S8) [29–31]. Though absolute expression level differed, eight of the nine genes showed an increased expression in materials of shoot apical meristem (Additional file 11: Figure S3), suggesting that these genes might play important roles during the development or environmental adaptation of roses.

### Characteristics of the *Rosa*-specific transcripts

**Fig. 5:**
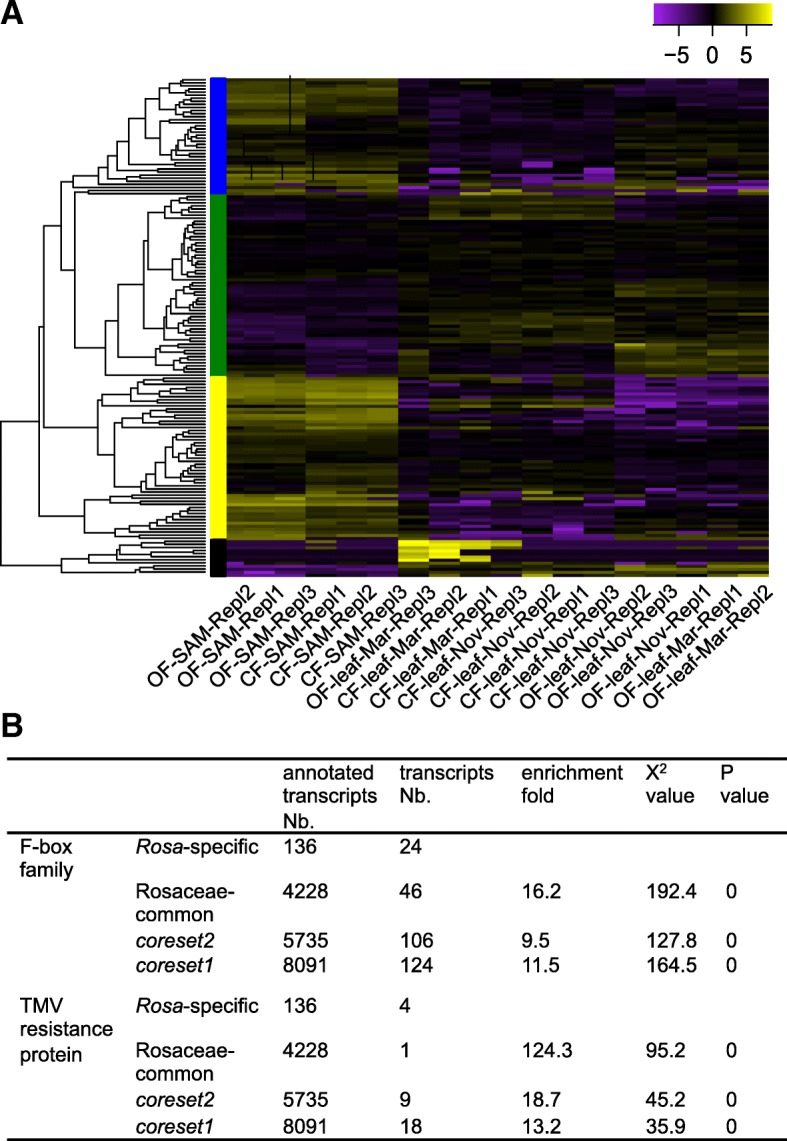
Identification and characterization *Rosa*-specific transcripts. **a** Heat map comparing scaled expression values for the 164 *Rosa*-specific transcripts. Yellow indicates higher while purple marks lower expression. Blue, green, yellow, and black bars indicate membership in the identified transcription clusters. **b** F-box and TMV resistance protein were significantly enriched in *Rosa*-specific transcripts. X^2^ tests were performed online (http://www.quantpsy.org/chisq/chisq.htm) by comparing the *Rosa*-specific transcripts number with those from Rosaceae-common, *coreset1* and *coreset2* genes. *P* values were corrected with Bonferroni correction

Among the 164 *Rosa*-specific molecules, 147 had protein-encoding potential with 136 of them having annotation (65 were uncharacterized previously; Additional file 12: Table S9). About 72 of these *Rosa*-specific genes were expressed at relatively lower level in both leaf and SAM materials (Fig. 5a, green and black bars), while around 92 of them (~ 56.1%) were expressed higher in SAMs than leaf materials (Fig. 5a, blue and yellow bars). No GO item was significant enriched. However, transcripts encoding for putative F-box family members were strongly enriched (24x; Fig. 5b; Additional file 12: Table S9). F-box proteins represent one of the largest super-families in eukaryotic organisms, and contain F-box motif to recognize substrate proteins in ubiquitin-proteasome pathway [32, 33]. F-box proteins play a pivotal role in many physiological activities such as cell-cycle progression, transcriptional regulation, hormone signaling, programmed cell death and cell signaling transduction. F-box proteins can be distinguished to three classifications: F-box/kelch-repeat type, F-box/LRR-repeat proteins, and other F-box proteins. At least seven *Rosa*-specific transcripts belonged to F-box/LRR-repeat type, which might play essentials roles in adaptation of biotic stresses (Additional file 12: Table S9). Coincidently, four TMV resistant genes, which are essential for defenses against various plant viruses [34, 35], were also significantly enriched in these *Rosa*-specific genes (Fig. 5b). These indicate that roses might have evolved new defense-related proteins to against its specific biotic stresses. Further experiments will be required to address the biological roles of these *Rosa*-specific transcripts.

## Discussion

As one of the most important horticultural plants, rose has its special biology. Continuous flowering, fragrance, flower shape, thorn and many traits not presenting in *Populus* and other woody model plants could be found in roses, hence roses are now becoming a model woody species for understanding the molecular mechanisms regulating these traits. Interestingly, the breeding of modern roses often involves frequent hybridization and polyploidization among species, which often feature stronger diseases resistance and cold resistance, better fragrance and lack of prickles [36–38]. On the other hand, tracing the processes and history of modern roses domestication and breeding remains a challenge [2].

In this report, we produced high quality transcriptome assemblies for *R. chinensis* ‘Old Blush’ (OB) and *R. wichuriana* ‘Bayes’ Thornless’ (BT) with transcripts N50 above 2 kb and mean length about 1.3 kb. By incorporating published data for OB, we generated an improved assembly with mean transcript length longer than 1.3 kb. We identified 10,073 highly conserved transcripts (*coreset1*) between OB and BT with BUSCO assay confirming the high level of completeness with these assemblies. As *coreset1* transcripts were based on a relatively stringent level of sequence identity (90%), they could directly be used to evaluate the differential expression of orthologous genes between species.

These assemblies were then explored to identify about 5959 transcripts shared by *Rosa sp*. Finally we detected 4447 transcripts shared among Rosaceae, and about 164 transcripts only present in the *Rosa* transcriptomes. Since comparative selection pressure analysis provides an efficient way for understanding the molecular signatures underlying morphological trait variation and domestication [39–41], we next identified the genes under positive selection in roses among these Rosaceae-common transcripts. This analysis detected 409 rose genes significantly selected with around 40% of them being highly expressed in SAM tissues, suggesting that they might play important roles in the development of young organs/tissues of roses. It’s noteworthy that nine of these positively selected genes belong to GO items related to DNA repair and stress adaptation (Fig. 4) [29–31, 42]. This strong enrichment seems be associated with a strong requirement in the biological processes upon genome damage/confliction due to switch of environmental conditions and/or frequent intra- and inter-species hybridization. The Rosaceae-common dataset contains about 504 single-copy transcripts overlapped with the BUSCO set of genes. These single-copy transcripts could be directly used to clarify the Rosaceae phylogenetic relationship (Fig. 4), a challenge likely caused by frequent hybridization, rapid radiation, polyploidization and domestication [43–47].

In contrast to the Rosaceae-common transcripts, the identification of *Rosa*-specific transcripts seemed very interesting. Although the domestication processes had been documented [2], the evolutionary history and molecular mechanisms controlling traits special for roses are still not clear [1]. About half of the 164 transcripts are uncharacterized or without known GO annotation. These *Rosa*-specific transcripts might be related to the phenotypes that have not been characterized in other species. It’s possible that sequences of these transcripts have significantly diverged but remain similar functions like the homologs in Arabidopsis and roses. Interestingly, we observed a strong enrichment of F-box and TMV resistant proteins. These proteins might play a role in processes involved in biotic stress adaptation of roses.

## Conclusions

In this study, we provided better quality transcriptome assemblies for roses, and pinned out the genes might make rose special. In addition, we identified transcripts common in Rosaceae, and these should help us to clarify the phylogenetic relationships of Rosaceae plants.

## Methods

### Plant materials and data generation

*R. chinensis* ‘Old Blush’ (OB) and *R. wichuriana* ‘Basye’s Thornless’ (BT) plants were grown in the glasshouses at the Flower Research Institute of Yunnan Academy of Agricultural Sciences (Kunming, Yunnan, China). Leaf materials of about 4 cm length (3.5–4.5 cm from base of pedicel to leaf tip with all the leaflets just became flatten; leaves at this stage were supposed to become completely functional for photosynthesis; Fig. 1) were collected in autumn (November 21st, 2015) from blooming OB plants and non-blooming BT, in spring (March 21st, 2016) when both species were setting flower buds (Fig. 1). Shoot tip materials with most leaf materials removed were sampled on 21st March, 2016. At least six biological replicates, composed each of at least 3 individual plants, were performed for each developmental stage and for each species.

Total RNA was isolated using the RNAprep Pure Plant Kit (Tiangen, Beijing) and mRNA was purified with poly-T oligo-attached magnetic beads (see Fig. 2 for workflow). Around 1 μg mRNA with RNA Integrity number (RIN) score greater than 8 was used for library construction. Fragmentation was carried out using divalent cations under elevated temperature in an Illumina proprietary fragmentation buffer. Sequencing libraries were generated using the TruSeq RNA Sample Preparation Kit (Illumina, San Diego, CA, USA). First strand cDNA was synthesized using random oligonucleotides and SuperScript II. Second strand cDNA synthesis was subsequently performed using DNA Polymerase I and RNase H. Remaining overhangs were converted into blunt ends via exonuclease/polymerase activities and the enzymes were removed. After adenylation of the 3′ ends of the DNA fragments, Illumina PE adapter oligonucleotides were ligated to prepare for hybridization. To select cDNA fragments of the preferred 380 bp in length, the library fragments were purified using the AMPure XP system (Beckman Coulter, Beverly, CA, USA). DNA fragments with ligated adaptor molecules on both ends were selectively enriched using Illumina PCR Primer Cocktail in a 15 cycle PCR reaction. Products were purified (AMPure XP system) and quantified using the Agilent high sensitivity DNA assay on a Bioanalyzer 2100 system (Agilent). Sequencing was carried out on either Illumina NextSeq 500 or Hiseq2000 platform.

### Data filtering

Approximately 105Gb pair-end data was generated for all samples (about 52.3Gb for BT and 52.8Gb for OB; Table 1). The final data volume for OB was about 116.1Gb including the published data. Data information for other species/materials was listed in Table 2. The quality of raw reads was assessed and filtered with a custom pipeline using FastQC (V0.10.1; http://www.bioinformatics.babraham.ac.uk/projects/fastqc) and Trimmomatic (V0.36; ILLUMINACLIP:TruSeq3-PE.fa:2:45:10/ LEADING:10/ TRAILING:10/SLIDINGWINDOW:4:25/MINLEN:48) [48]. Adaptor sequences, reads PHRED quality below 92%, and PCR duplicates were all removed with custom perl scripts (https://github.com/ckenkel/annotatingTranscriptomes). Short read archive (SRA) accessions for all data are found in Table 1.

### Assembly and functional annotation of transcriptomes for OB and BT

Prior to assembly, data for each species was concatenated (SAM, leaf_Nov and leaf_Mar), and read abundance was normalized to 50x coverage using the in silico normalization tool in Trinity [49] to spare assembly time and minimize memory requirements. Assembly for BT was constructed with data generated from this study, while two assemblies were built up first with the newly produced data and later combining with published data (see Tables 1 and 2). After filtering and normalization the data was about 157 Gb, comprising approximately 1.3 billion normalized read pairs, which were then assembled using optimized parameters (Kmer = 2, min_glue = 5, SS_lib RF) in *Trinity* (r2014_07–17) [49]. Trinity assembly was clustered by TGICL toolkit to remove identified duplicates [50]. Open reading frames (ORFs) were predicted using *Transdecoder* (https://github.com/TransDecoder/TransDecoder/wiki). *Hmmer3* was used to identify additional ORFs matching *Pfam-A* domains [51].

The completeness of these assemblies was further evaluated with Benchmarking Universal Single-Copy Orthologs (BUSCO) strategy with RNA mode using 1440 near-universal single-copy orthologs [52]. Functional annotation was performed for each of the transcriptomes at the peptide level using a custom pipeline that defines protein products and assigns transcript names against multiple databases (Fig. 2). Predicted proteins/peptides were analyzed using InterProScan5 [53], which searched all available databases including Gene Ontology (GO:201605). BLASTx analysis was performed with the NCBI non-redundant protein sequences (NR) database, eukaryotic ortholog groups (KOG) database, KEGG ortholog (KO) database, Swiss-Prot protein database, Gene Ontology (GO) database, and protein family (PFAM) database. The resulting *.gff3* and *.tbl* files were further annotated with functional descriptors in Transvestigator (10.5281/zenodo.10471).

### Calling of the conserved orthologous transcript elements set between OB and BT (*coreset1*), and for *Rosa* (*coreset2*)

To identify the transcripts shared between the two *Rosa* species, we identified *coreset1* between OB and BT using orthoMCL [54] and an optimized reciprocal blast method [55] with a sequence identity at 90%, coverage above 0.7 and more than 200 bp length.

To screen for shared transcripts within *Rosa* (*coreset2*), we compared *coreset1* to other published RNA-seq data for *R. multiflora*, *R. roxburghii*, etc. (see Table 2 for the data used in this analysis). Sequence identity was set at 80%.

### Identification of Rosaceae-common and *Rosa*-specific transcripts

We further compared the *coreset2* transcripts to known CDS for *Malus domestica* (v3.0.a1), *Prunus avium* (v1.0.a1), *Rubus occidentalis* (v1.0.a1), *Fragaria veseca* (v4.0) in order to find out the transcripts present in all Rosaceae and only in *Rosa* plants. Reciprocal blast analysis was carried out at sequence identity of 70% with other parameters the same like for coreset1. As the *coreset2* transcripts were used to blast against the CDS from other plants, this analysis did not detect other genera-specific transcripts/genes. Both sets of transcripts were later subjected to GO and KEGG Orthology (KO) enrichment analysis on the basis of a hypergeometric test (FDR < 0.05).

### Selection analysis of Rosaceae-common transcripts

Selection pressure analysis was performed for the 4341 transcripts with coding potential in Rosaceae-common set of molecules as previously described [39–41]. Putative orthologous genes from *M. domestica, P. avium* and *F. veseca* were treated as background, while *Rosa* transcripts were considered as foreground for the selection analysis. Amino acid alignments were carried out with *ClustalO* (with default parameters) [56]. Phylogenetic analysis was done with *FastTree* (*−gtr –gamma*; http://tree.bio.ed.ac.uk/ software/figtree/). The *codeml* program in *PAML* package was used to estimate the synonymous (*dS*) and non-synonymous (*dN*) rates in these transcripts according to Yang [57]. *Fisher’s exact test* with Bonferroni correction was used to compare the significance of selection pressure between H0 and H1 models.

## Additional files

Additional file 1: **Table S1.** BUSCO analysis of the assemblies and *coreset1*. C, S, D, F, and M represent the proportion of the complete, the complete and single-copy, the complete and duplicated, the fragmented, and the missing BUSCO transcripts, respectively. (XLSX 128 kb)

Additional file 2: **Table S2.** Annotation details for both genotypes. COG, GO, KEGG, KOG, Pfam, Swissprot, TrEMBL, NR and NT database were used in the annotation process. (XLS 24958 kb)

Additional file 3: **Figure S1.** Annotation results to Nr database. A for BT and B for OB. Transcripts of BT and OB were blasted to NR database using default parameters with the best hit kept. Colors indicate the annotation proportion for each closet species. The highest proportion of best hit for both genotypes is *Fragaria vesca* (around 75%), while *Prunus mune* occupies the second highest proportion. (PDF 251 kb)

Additional file 4: **Table S3.** KEGG and GO enrichment analysis for *coreset1* transcripts. Note that each transcript could be mapped to several GO terms and KEGG categories. (XLSX 306 kb)

Additional file 5: **Table S4.** KEGG and GO clustering information of 4447 Rosaceae-common genes. (XLSX 274 kb)

Additional file 6: **Table S5.** List of the Rosaceae-common single-copy transcripts. (XLSX 15 kb)

Additional file 7: **Figure S2.** Phylogenetic clustering of the four species based on the 4447 Rosaceae-common transcripts. For each transcript, a Neighbor-Joining tree was constructed. The topologies in A, B, and C show the phylogenetic relationship supported by 65 and 33% (A), 0.7 and 0.4% (B), 0.5 and 0.5% (C) of transcripts. (PDF 34 kb)

Additional file 8: **Table S6.** Raw data for the positively selected genes in the Rosaceae-common transcripts. (XLSX 185 kb)

Additional file 9: **Table S7.** GO annotation for the 409 positively selected genes. (XLSX 22 kb)

Additional file 10: **Table S8.** Detailed annotation and expression levels for the nine positively selected genes with significant GO-enrichment. (XLSX 15 kb)

Additional file 11: **Figure S3.** Clustered heat map of expression for the nine selected Rosacaeae-common transcripts. Red (high) and blue (low) mark the expression levels. See Figs. 4 and 5 for further information. (PDF 5 kb)

Additional file 12: **Table S9.** Detailed information for the 164 *Rosa*-specific genes. (XLSX 20 kb)

## Abbreviations

BT: *Rosa wichuriana* ‘Basye’s Thornless’
BUSCO: Benchmarking Universal Single-Copy Orthologs
Coreset: Conserved Orthologous Transcript Elements Sets
dN: Non-synonymous change
dS: Synonymous change
GO: Gene Ontology
OB: *Rosa chinensis* ‘Old Blush’
RIN: RNA Integrity number
SAM: Shoot apical meristem

## References

[1] Dong X, Jiang X, Kuang G, Wang Q, Zhong M, Jin D, Hu J-Y (2017). Genetic control of flowering time in woody plants: roses as an emerging model. Plant Diversity.

[2] Bendahmane M, Dubois A, Raymond O, Bris ML (2013). Genetics and genomics of flower initiation and development in roses. J Exp Bot.

[3] Li S, Zhou N, Zhou Q, Yan H, Jian H, Wang Q, Chen M, Qiu X, Zhang H, Wang S (2015). Inheritance of perpetual blooming in *Rosa chinensis* ‘old blush’. Horticult Plant J.

[4] Randoux M, Daviere JM, Jeauffre J, Thouroude T, Pierre S, Toualbia Y, Perrotte J, Reynoird JP, Jammes MJ, Oyant LHS (2014). RoKSN, a floral repressor, forms protein complexes with RoFD and RoFT to regulate vegetative and reproductive development in rose. New Phytol.

[5] Iwata H, Gaston A, Remay A, Thouroude T, Jeauffre J, Kawamura K, Oyant LH-S, Araki T, Denoyes B, Foucher F (2012). The TFL1 homologue KSN is a regulator of continuous flowering in rose and strawberry. Plant J.

[6] de Vries DP, Dubois LAM (1996). Rose breeding: past, present, prospects.

[7] Debener T, Linde M (2009). Exploring complex ornamental genomes: the rose as a model plant. Crit Rev Plant Sci.

[8] Vukosavljev M, Zhang J, Esselink GD, van ‘t Westende WPC, Cox P, Visser RGF, Arens P, Smulders MJM (2013). Genetic diversity and differentiation in roses: a garden rose perspective. Sci Hortic.

[9] Li DGS, Zhao H, Wang X (2016). Research advances in the genetic relationship of rose germplasm resources. J Biol.

[10] Zhu Z-M, Gao X-F, Fougère-Danezan M (2015). Phylogeny of Rosa sections Chinenses and Synstylae (Rosaceae) based on chloroplast and nuclear markers. Mol Phylogenet Evol.

[11] Fougere-Danezan M, Joly S, Bruneau A, Gao X-F, Zhang L-B (2015). Phylogeny and biogeography of wild roses with specific attention to polyploids. Ann Bot.

[12] Koopman WJ, Volker W,Katrien DC, Johan VH, Jan DR, JH SG, Dirk V, Ben V, M RC, Bert M et al. AFLP markers as a tool to reconstruct complex relationships: a case study in Rosa (Rosaceae). Am J Bot. 2008;95(3):353–66. 10.3732/ajb.95.3.353 21632360

[13] Cairns T (2007). Modern roses XII.

[14] Foucher F, Hibrand-Saint Oyant L, Hamama L, Sakr S, Nybom H, Baudino S, Caissard JP, Hokanson SC, Byrne DM, Smulder JMS, Debener T, Linde M (2015). Towards the Rose Genome Sequence and Its Use in Research and Breeding. Vi International Symposium on Rose Research and Cultivation.

[15] Raymond O, Gouzy J, Just J, Badouin H, Verdenaud M, Lemainque A, Vergne P, Moja S, Choisne N, Pont C, et al. The Rosa genome provides new insights into the domestication of modern roses. Nat Genet. 2018;50:772–77. 10.1038/s41588-018-0110-3 PMC5984618 29713014

[16] Guterman I, Shalit M, Menda N, Piestun D, Dafny-Yelin M, Shalev G, Bar E, Davydov O, Ovadis M, Emanuel M (2002). Rose scent: genomics approach to discovering novel floral fragrance–related genes. Plant Cell.

[17] Dubois A, Remay A, Raymond O, Balzergue S, Chauvet A, Maene M, Pécrix Y, Yang S-H, Jeauffre J, Thouroude T (2011). Genomic approach to study floral development genes in Rosa sp. PLoS One.

[18] Dubois A, Carrere S, Raymond O, Pouvreau B, Cottret L, Roccia A, Onesto J-P, Sakr S, Atanassova R, Baudino S (2012). Transcriptome database resource and gene expression atlas for the rose. BMC Genomics.

[19] Yan H, Hao Z, Min C, Jian H, Baudino S, Caissard JC, Bendahmane M, Li S, Zhang T, Zhou N (2014). Transcriptome and gene expression analysis during flower blooming in Rosa chinensis ‘pallida’. Gene.

[20] Yan H, Zhang H, Wang Q, Jian H, Qiu X, Baudino S, Just J, Raymond O, Gu L, Wang J (2016). The Rosa chinensis cv. Viridiflora Phyllody Phenotype is associated with misexpression of flower organ identity genes. Front Plant Sci.

[21] Zhang XY, Zhang JZ, Zhang WW, Yang T, Xiong Y, Che DD (2016). Transcriptome sequencing and de novo analysis of Rosa multiflora under cold stress. Acta Physiol Plant.

[22] Gao Y, Liu C, Li X, Xu H, Liang Y, Ma N, Fei Z, Gao J, Jiang C-Z, Ma C (2016). Transcriptome profiling of petal abscission zone and functional analysis of an Aux/IAA family gene RhIAA16 involved in petal shedding in rose. Front Plant Sci.

[23] Vukosavljev M, Arens P, Voorrips RE, van ‘t Westende WPC, Esselink GD, Bourke PM, Cox P, van de Weg WE, Visser RGF, Maliepaard C (2016). High-density SNP-based genetic maps for the parents of an outcrossed and a selfed tetraploid garden rose cross, inferred from admixed progeny using the 68k rose SNP array. Horticult Res.

[24] Koning-Boucoiran CFS, Esselink GD, Vukosavljev M, van ‘t Westende WPC, Gitonga VW, Krens FA, Voorrips RE, van de Weg WE, Schulz D, Debener T (2015). Using RNA-Seq to assemble a rose transcriptome with more than 13,000 full-length expressed genes and to develop the WagRhSNP 68k Axiom SNP array for rose (Rosa L.). Front Plant Sci.

[25] Hibrand Saint-Oyant L, Ruttink T, Hamama L, Kirov I, Lakhwani D, Zhou NN, Bourke PM, Daccord N, Leus L, Schulz D, et al. A high-quality genome sequence of Rosa chinensis to elucidate ornamental traits. Nat Plants. 2018;4:1–16. 10.1038/s41477-018-0166-1 PMC6786968 29892093

[26] Guo X, Yu C, Luo L, Wan H, Zhen N, Xu T, Tan J, Pan H, Zhang Q (2017). Transcriptome of the floral transition in Rosa chinensis ‘Old Blush’. BMC Genomics.

[27] Han Y, Wan H, Cheng T, Wang J, Yang W, Pan H, Zhang Q (2017). Comparative RNA-seq analysis of transcriptome dynamics during petal development in Rosa chinensis. Sci Rep.

[28] Yan X, Zhang X, Lu M, He Y, An H (2015). De novo sequencing analysis of the Rosa roxburghii fruit transcriptome reveals putative ascorbate biosynthetic genes and EST-SSR markers. Gene.

[29] Rohde A, Morreel K, Ralph J, Goeminne G, Hostyn V, De Rycke R, Kushnir S, Van Doorsselaere J, Joseleau J-P, Vuylsteke M (2004). Molecular phenotyping of the <em>pal1</em> and <em>pal2</em> mutants of <em>Arabidopsis thaliana</em> reveals far-reaching consequences on Phenylpropanoid, amino acid, and carbohydrate metabolism. Plant Cell.

[30] Rowan BA, Oldenburg DJ, Bendich AJ (2010). RecA maintains the integrity of chloroplast DNA molecules in Arabidopsis. J Exp Bot.

[31] Stroud H, Greenberg Maxim VC, Feng S, Bernatavichute Yana V, Jacobsen Steven E (2013). Comprehensive analysis of silencing mutants reveals complex regulation of the Arabidopsis Methylome. Cell.

[32] Wei C, Li H, Tian M, Yu X, Liu D (2017). Research Progress for F-box protein family function in Arabidopsis thaliana. Acta Botan Boreali-Occiden Sin.

[33] Stefanowicz K, Lannoo N, Van Damme EJM (2015). Plant F-box proteins - judges between life and death. Crit Rev Plant Sci.

[34] Wilson TMA (1993). Strategies to protect crop plants against viruses - pathogen-derived resistance blossoms. P Natl Acad Sci USA.

[35] Simon-Mateo C, Garcia JA (2011). Antiviral strategies in plants based on RNA silencing. Bba-Gene Regul Mech.

[36] Liu CY, Wang GL, Wang H, Xia T, Zhang SZ, Wang QG, Fang YM (2015). Phylogenetic relationships in the genus Rosa revisited based on rpl16, trnL-F, and atpB-rbcL sequences. Hortscience.

[37] Liu Y, Liu Q (2004). Evaluation and exploitation of genetic resources in roses. J Plant Genet Resour.

[38] Qiu X, Zhang H, Li S, Jian H, Tang K (2009). The relative relationships analysis of rose germplasm in Yunnan based on SSR. Acta Botan Boreali-Occiden Sin.

[39] Khan MR, Hu J-Y, Riss S, He C, Saedler H (2009). MPF2-like-a MADS-box genes control the inflated Calyx syndrome in Withania (Solanaceae): roles of Darwinian selection. Mol Biol Evol.

[40] Peng Z, Lu Y, Li L, Zhao Q, Feng Q, Gao Z, Lu H, Hu T, Yao N, Liu K (2013). The draft genome of the fast-growing non-timber forest species moso bamboo (Phyllostachys heterocycla). Nat Genet.

[41] Koenig D, Jimenez-Gomez JM, Kimura S, Fulop D, Chitwood DH, Headland LR, Kumar R, Covington MF, Devisetty UK, Tat AV (2013). Comparative transcriptomics reveals patterns of selection in domesticated and wild tomato. P Natl Acad Sci USA.

[42] Li J-T, Gao Y-D, Xie L, Deng C, Shi P, Guan M-L, Huang S, Ren J-L, Wu D-D, Ding L (2018). Comparative genomic investigation of high-elevation adaptation in ectothermic snakes. P Natl Acad Sci USA.

[43] Xiang Y, Huang C-H, Hu Y, Wen J, Li S, Yi T, Chen H, Xiang J, Ma H (2017). Evolution of Rosaceae fruit types based on nuclear phylogeny in the context of geological times and genome duplication. Mol Biol Evol.

[44] Zhang S-D, Jin J-J, Chen S-Y, Chase MW, Soltis DE, Li H-T, Yang J-B, Li D-Z, Yi T-S (2017). Diversification of Rosaceae since the late cretaceous based on plastid phylogenomics. New Phytol.

[45] Potter D, Eriksson T, Evans RC, Oh S, Smedmark JEE, Morgan DR, Kerr M, Robertson KR, Arsenault M, Dickinson TA (2007). Phylogeny and classification of Rosaceae. Plant Syst Evol.

[46] Campbell CS, Evans RC, Morgan DR, Dickinson TA, Arsenault MP (2007). Phylogeny of subtribe Pyrinae (formerly the Maloideae, Rosaceae): limited resolution of a complex evolutionary history. Plant Syst Evol.

[47] David RM, Douglas ES, Kenneth RR (1994). Systematic and evolutionary implications of rbcL sequence variation in Rosaceae. Am J Bot.

[48] Bolger AM, Lohse M, Usadel B (2014). Trimmomatic: a flexible trimmer for Illumina sequence data. Bioinformatics.

[49] Grabherr MG, Haas BJ, Yassour M, Levin JZ, Thompson DA, Amit I, Adiconis X, Fan L, Raychowdhury R, Zeng Q (2011). Full-length transcriptome assembly from RNA-Seq data without a reference genome. Nat Biotech.

[50] Pertea G, Huang X, Liang F, Antonescu V, Sultana R, Karamycheva S, Lee Y, White J, Cheung F, Parvizi B (2003). TIGR gene indices clustering tools (TGICL): a software system for fast clustering of large EST datasets. Bioinformatics.

[51] Mistry J, Finn RD, Eddy SR, Bateman A, Punta M (2013). Challenges in homology search: HMMER3 and convergent evolution of coiled-coil regions. Nucleic Acids Res.

[52] Simão FA, Waterhouse RM, Ioannidis P, Kriventseva EV, Zdobnov EM (2015). BUSCO: assessing genome assembly and annotation completeness with single-copy orthologs. Bioinformatics.

[53] Jones P, Binns D, Chang H-Y, Fraser M, Li W, McAnulla C, McWilliam H, Maslen J, Mitchell A, Nuka G (2014). InterProScan 5: genome-scale protein function classification. Bioinformatics.

[54] Li L, Stoeckert CJ, Roos DS (2003). OrthoMCL: identification of ortholog groups for eukaryotic genomes. Genome Res.

[55] Moreno-Hagelsieb G, Latimer K (2008). Choosing BLAST options for better detection of orthologs as reciprocal best hits. Bioinformatics.

[56] Sievers F, Wilm A, Dineen D, Gibson TJ, Karplus K, Li WZ, Lopez R, McWilliam H, Remmert M, Soding J (2011). Fast, scalable generation of high-quality protein multiple sequence alignments using Clustal omega. Mol Syst Biol.

[57] Yang Z (2007). PAML 4: phylogenetic analysis by maximum likelihood. Mol Biol Evol.

