# Supplementary figures and images for: Comparative transcriptomics identifies patterns of selection in roses
Source: BMC Plant Biol. 2018 Dec 22;18:371. doi: 10.1186/s12870-018-1585-x (PMC6303930; doi:10.1186/s12870-018-1585-x)

**A**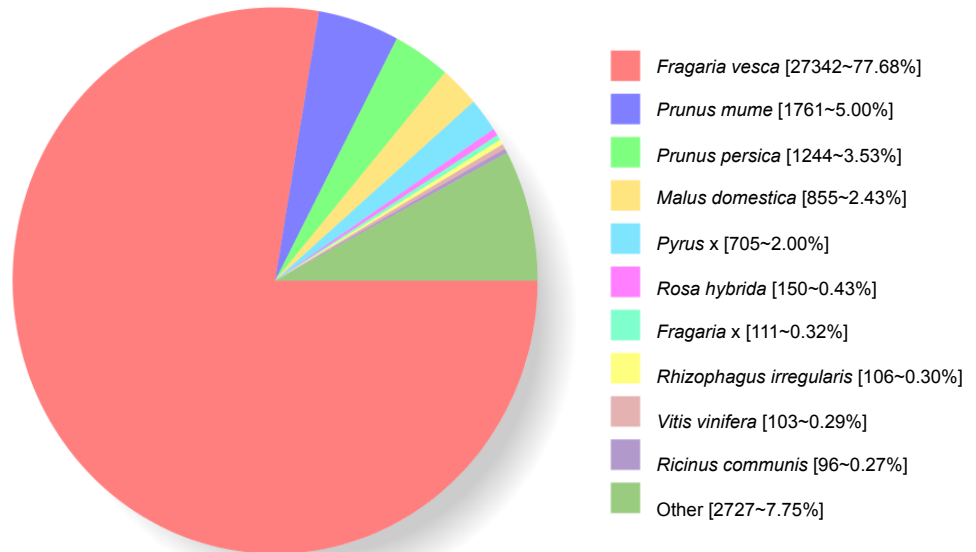**B**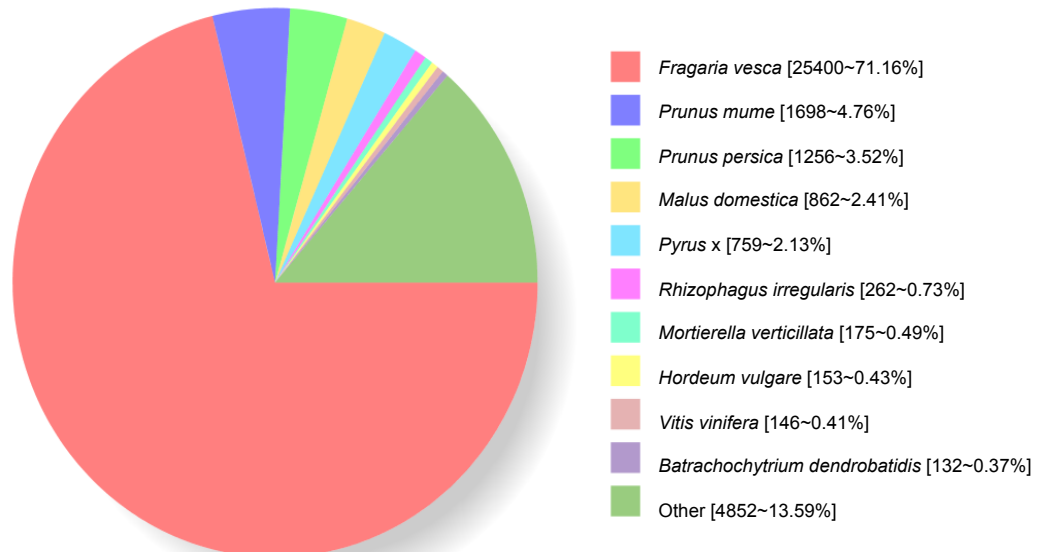

Supplement: Supplementary file 3 — Figure S1. Annotation results to Nr database. A for BT and B for OB. Transcripts of BT and OB were blasted to NR database using default parameters with the best hit kept. Colors indicate the annotation proportion for each closet species. The highest proportion of best hit for both genotypes is Fragaria vesca (around 75%), while Prunus mune occupies the second highest proportion. (PDF 251 kb) [file 12870_2018_1585_MOESM3_ESM.pdf]

**A**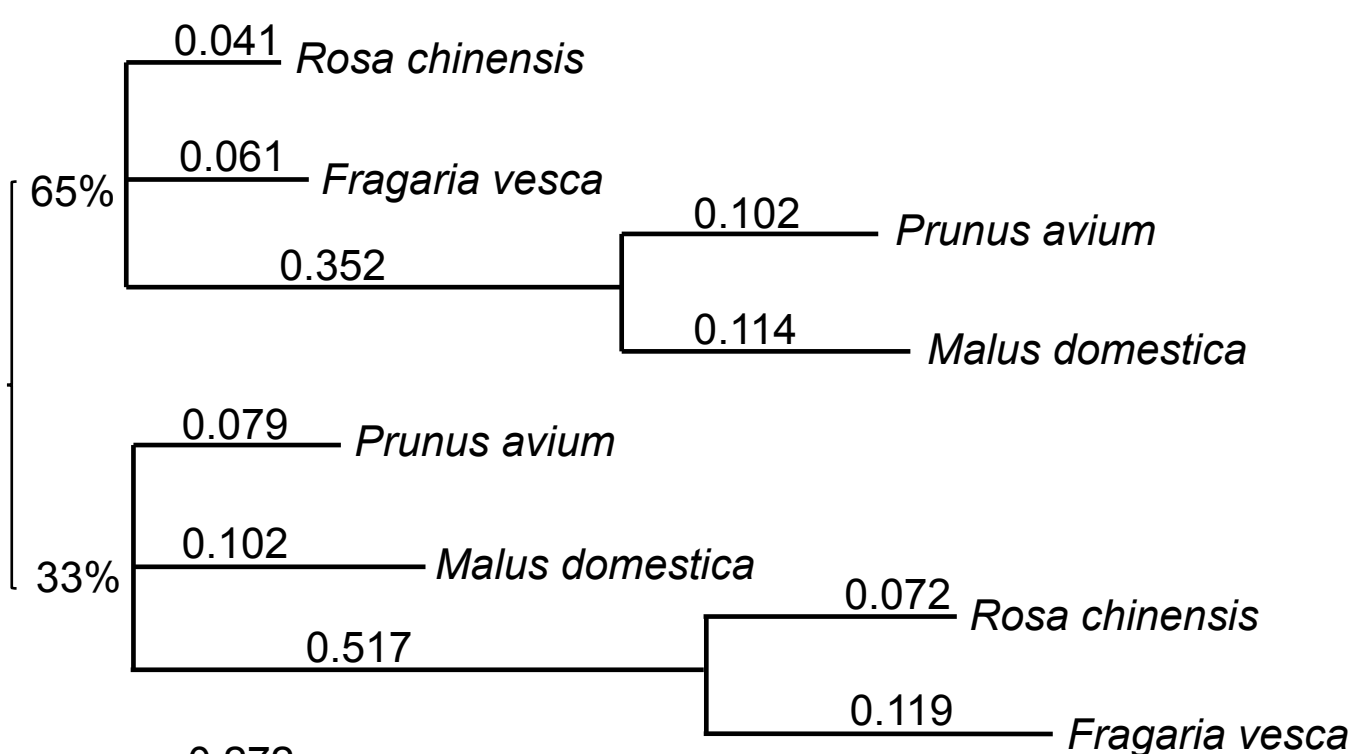**B**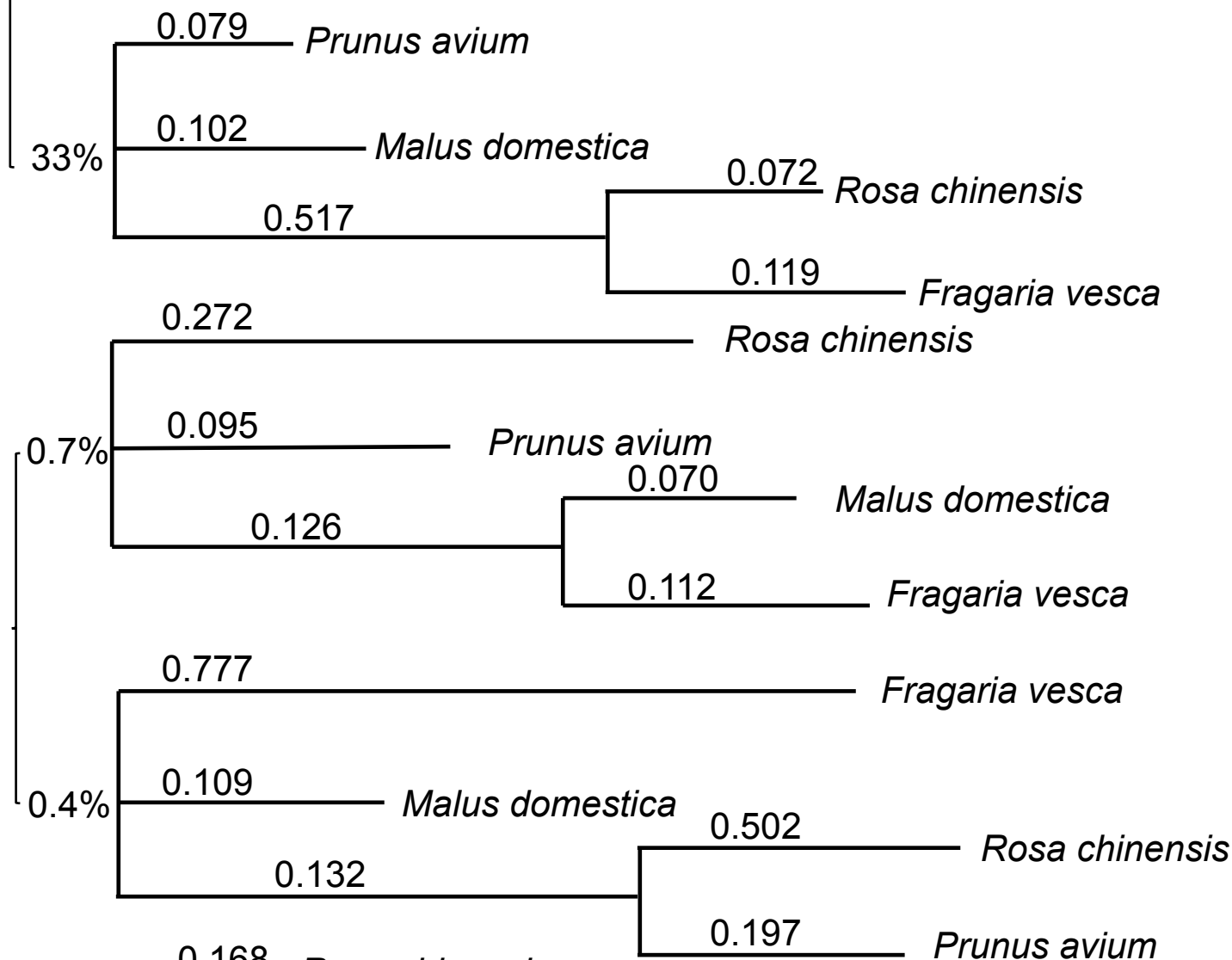**C**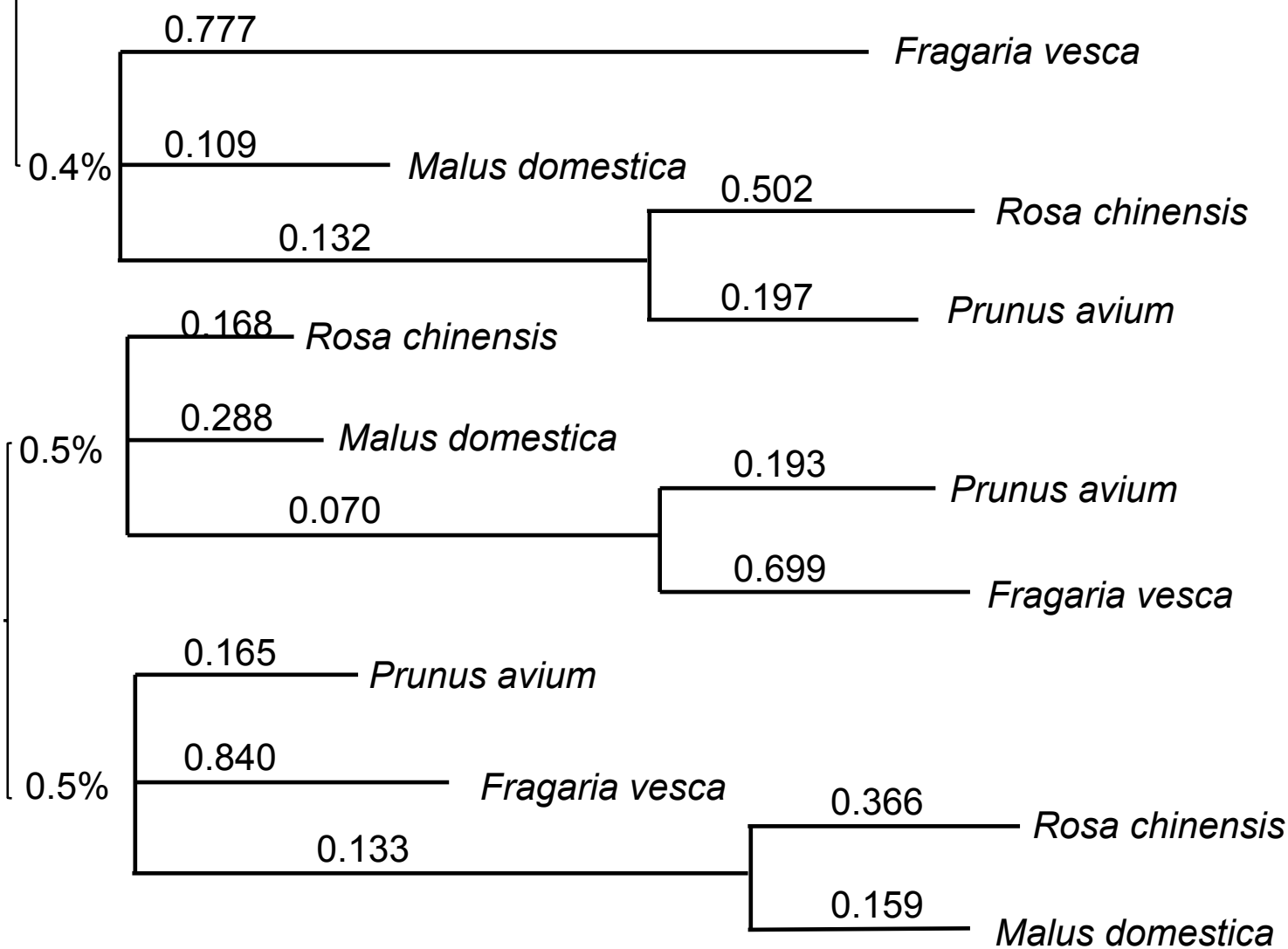

Supplement: Supplementary file 7 — Figure S2. Phylogenetic clustering of the four species based on the 4447 Rosaceae-common transcripts. For each transcript, a Neighbor-Joining tree was constructed. The topologies in A, B, and C show the phylogenetic relationship supported by 65 and 33% (A), 0.7 and 0.4% (B), 0.5 and 0.5% (C) of transcripts. (PDF 34 kb) [file 12870_2018_1585_MOESM7_ESM.pdf]

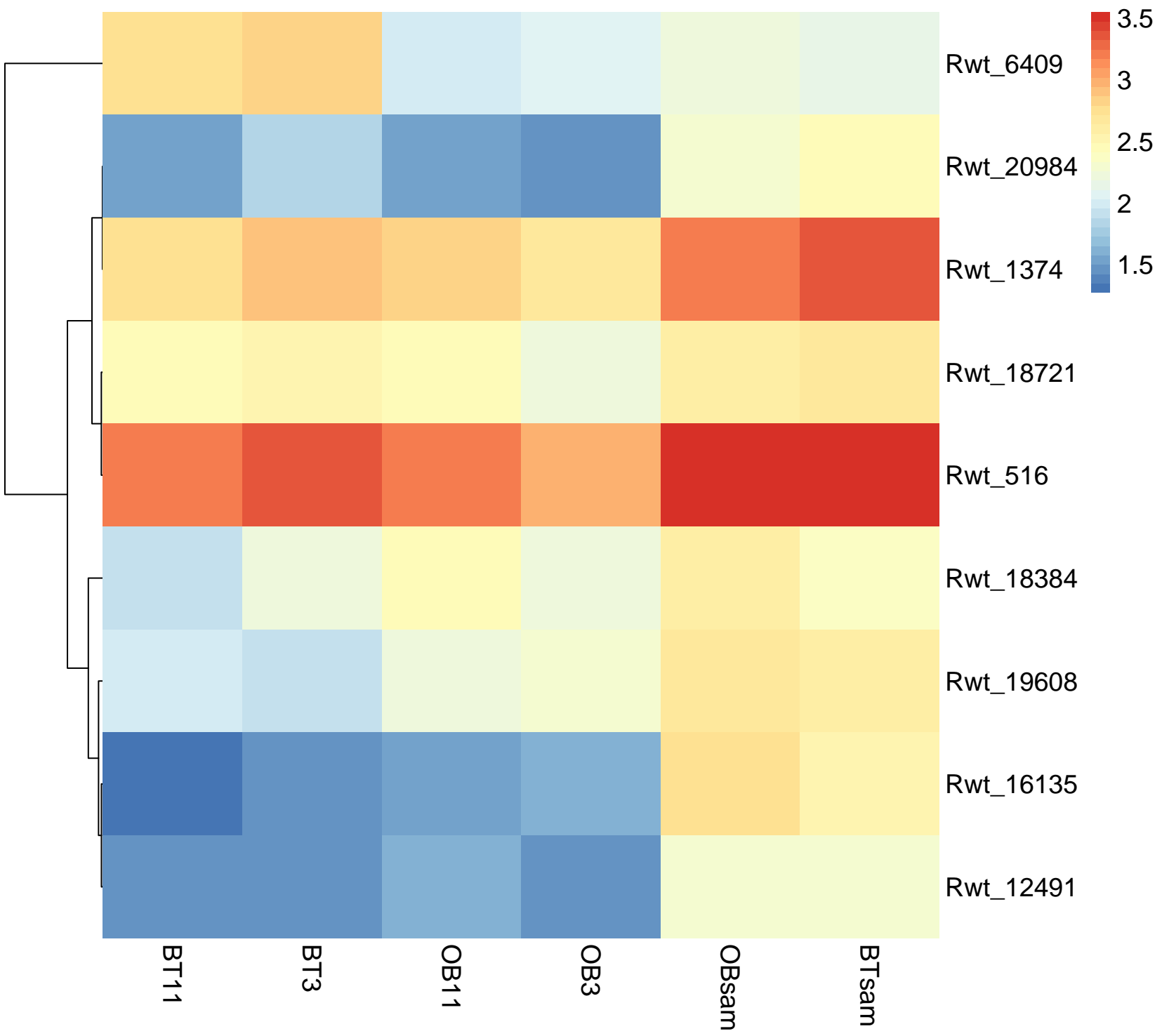

Supplement: Supplementary file 11 — Figure S3. Clustered heat map of expression for the nine selected Rosacaeae-common transcripts. Red (high) and blue (low) mark the expression levels. See Figs. 4 and 5 for further information. (PDF 5 kb) [file 12870_2018_1585_MOESM11_ESM.pdf]
